# PROX1 and β-catenin are prognostic markers in pancreatic ductal adenocarcinoma

**DOI:** 10.1186/s12885-016-2497-5

**Published:** 2016-07-13

**Authors:** Kapo Saukkonen, Jaana Hagström, Harri Mustonen, Anne Juuti, Stig Nordling, Pauliina Kallio, Kari Alitalo, Hanna Seppänen, Caj Haglund

**Affiliations:** Department of Surgery, University of Helsinki and Helsinki University Hospital, P.O. Box 440, FIN-00029 HUS Helsinki, Finland; Research Programs Unit, Translational Cancer Biology, University of Helsinki, P.O. Box 63, Helsinki, FIN-00014 Finland; Department of Pathology, Haartman Institute and HUSLAB, University of Helsinki and Helsinki University Hospital, Helsinki, FIN-00014 Finland

**Keywords:** Pancreatic ductal adenocarcinoma, Beta-catenin, PROX1, Prognosis

## Abstract

**Background:**

The Wnt/β-catenin pathway has a key role in regulating cellular processes and its aberrant signaling can lead to cancer development. The role of β-catenin expression in pancreatic ductal adenocarcinoma is somewhat controversial. Transcription factor PROX1 is a target of Wnt/β-catenin signaling and it is involved in carcinogenesis through alterations in its expression. The actions can be either oncogenic or tumor suppressive depending on the tissue. The aim of this study was to investigate PROX1 and β-catenin expression in pancreatic ductal adenocarcinoma (PDAC).

**Methods:**

Expression of PROX1 and β-catenin were evaluated in 156 patients by immunohistochemistry of tissue microarrays. Associations between tumor marker expression and clinicopathological parameters were assessed by the Fischer’s exact-test or the linear-by-linear association test. The Kaplan-Meier method and log-rank test were used for survival analysis. Uni- and multivariate survival analyses were carried out by the Cox regression proportional hazard model.

**Results:**

High PROX1 expression was seen in 74 (48 %) tumors, and high β-catenin expression in 100 (65 %). High β-catenin expression was associated with lower tumor grade (*p* = 0.025). High PROX1 and β-catenin expression associated significantly with lower risk of death from PDAC in multivariate analysis (HR = 0.63; 95 % CI 0.42–0.95, *p* = 0.026; and HR = 0.54; 95 % CI 0.35–0.82, *p* = 0.004; respectively). The combined high expression of PROX1 and β-catenin also predicted lower risk of death from PDAC (HR = 0.46; 95 % CI 0.28–0.76, *p* = 0.002).

**Conclusion:**

In conclusion, high PROX1 and β-catenin expression were independent factors for better prognosis in pancreatic ductal adenocarcinoma.

**Electronic supplementary material:**

The online version of this article (doi:10.1186/s12885-016-2497-5) contains supplementary material, which is available to authorized users.

## Background

The Wnt/β-catenin signaling pathway has a role in regulating cellular processes including organ development and differentiation, and tissue homeostasis in adults [1]. It is widely established that its aberrant signaling can lead to cancer development [2]. β-catenin is a key molecule in this pathway. It is an intracellular protein that is localized in cell membrane, cytoplasm and nucleus. The binding of Wnt ligand to its receptors inhibits β-catenin phosphorylation, which allows β-catenin to escape from degradation. It accumulates in the cytoplasm, and translocates to the nucleus. After localizing to the nucleus, β-catenin activates a target gene expression through interacting mainly with members of the T-cell factor/lymphoid enhancer factor (TCF/LEF) family of transcription factors (as reviewed in [3, 4]). In colorectal cancer (CRC), most tumors have a mutation in a key regulatory factor of the Wnt/β-catenin pathway. Often the mutation is in adenomatous polyposis coli *(APC)* or protein β-catenin encoding gene *(CTNNB1)*, which results in activation of the pathway [3].

In pancreatic ductal adenocarcinoma (PDAC), the role of the Wnt/β-catenin signaling pathway is controversial because of the variable and sometimes paradoxical effects in the pancreas. PDAC is a genetically heterogenous cancer with several key mutated genes including *KRAS2, CDKN2A/p16, SMAD4/DPC4,* and *TP53* [5]. Although genetic alterations of the Wnt signaling pathway are involved in PDAC tumors [6], mutations of *APC* or *CTNNB1* are less common [7]. Heiser et al. showed in mice that by introducing a β-catenin stabilizing mutation in *CTNNB1* leads to pancreatic hypoplasia at an early phase of the developing pancreas. If this mutation is introduced in later phase in the developing pancreas, it results in enlargement of the exocrine pancreas without tumor formation [8].

An immunohistochemically positive expression of β-catenin has been reported earlier, but the results have remained somewhat controversial. Lowy et al. noted reduced membranous expression of β-catenin in PDAC correlating with loss of tumor differentiation [9]. However, there is evidence that the Wnt/β-catenin signaling pathway is upregulated in PDAC both by immunohistochemistry and polymerase chain reaction [7, 10]. So far, the prognostic significance of β-catenin expression in PDAC has been investigated in a few studies with rather short follow-up times [11–14].

The transcription factor PROX1 has been shown to be a downstream target of the Wnt/β-catenin/TCF pathway in colorectal tumor neoplastic transformation and progression [15]. PROX1 is a transcriptional regulator and a part of the homeobox transcription factor family [16]. It has a key role in the development of the central nervous system [17], lens [18], liver [19], pancreas [19], lymphatic system [20], and heart [21]. But in addition, it is involved in oncogenesis through alterations in its expression. Depending on the tissue it can act either as a tumor suppressor or as an oncogene [22].

Recently, Wiener et al. constituted that PROX1 functions as a stem cell regulator in intestinal adenomas and in CRC, but not in the normal intestine [23]. In high-grade gliomas, and in colorectal cancer, high PROX1 tissue expression is associated with poor patient survival [24]. In esophageal squamous cell carcinoma PROX1 mediates the anti-proliferative effect by IFN-γ [25]. In hematological malignancies and in breast cancer PROX1 expression has been shown to be decreased [26, 27]. In hepatocellular carcinoma, depletion of PROX1 causes a significant increase in cell proliferation, and patients with high PROX1 expression have better prognosis compared to patients with low expression [28]. Schneider et al. showed that PROX1 is less expressed in pancreatic cancer cells than in the normal exocrine pancreas [29]. They also noticed that the gene expression level of PROX1 was lower in patients who survived less than 6 months than in patients with longer survival [29]. However, to our knowledge, immunohistochemical prognostic studies of PROX1 tissue expression are lacking in PDAC.

The aim of this study was to examine tumor expression and prognostic value of PROX1 and β-catenin in PDAC.

## Methods

### Patients

This study is based on a series of 189 consecutive PDAC patients surgically treated in 2000–2011 at the Department of Surgery, Helsinki University Hospital. Only patients with verified PDAC were included in this study. Median age at operation was 64 (range 39–84) years. Twenty-one patients, who received neoadjuvant chemotherapy, were excluded from the study. Eight patients were eventually diagnosed with stage IV disease with distant metastases according to the American Joint Committee on Cancer Pancreatic Cancer Staging System [30]**,** and four patients lacked data on stage. They were excluded from the study. Altogether, 156 patients were included in the study. Patients’ records, the Finnish Population Registry and Statistics Finland were used to obtain survival data and cause of death of the patients. A description of the study cohort is in Table 5.

### Preparation of tumor tissue microarrays and immunohistochemistry

Formalin-fixed and paraffin-embedded surgical tissue samples were collected from the archives of the Department of Pathology, Helsinki University Hospital. Experienced pathologists (J.H. and S.N.) re-evaluated all samples for confirmation of the histopathological diagnosis of PDAC. Representative regions of tumor specimens were defined and tumor areas were marked on hematoxylin- and eosin-stained tumor slides for preparation of tissue microarray blocks (TMA). Two 1.0-mm cores were taken from each tumor block with a semiautomatic tissue microarrayer (Tissue Arrayer 1, Beecher Instruments Inc., Silver Spring, MD, USA). In order to evaluate TMA representativeness compared to whole tissue blocks, we examined altogether six spots per patient taken from different areas/parts of the tumor.

TMA blocks were freshly cut into 4-μm sections. After deparaffinization in xylene and rehydration through a gradually decreasing concentration of ethanol to distilled water, slides were treated in a PreTreatment module (Lab Vision Corp., Fremont, CA, USA) in Tris–HCl (pH 8.5) and Tris-EDTA (pH 9) buffer for 20 min at 98 °C for antigen retrieval. Staining of sections was performed in an Autostainer 480 (Lab Vision Corp., Fremont, CA, USA) by the Dako REAL EnVision Detection system, Peroxidase/DAB+, Rabbit/Mouse (Dako, Glostrup, Denmark) for β-catenin, and by ImmPRESS HRP Polymer Detection Kit, Peroxidase, Anti-Goat IgG (Vector Laboratories, Burlingame, CA, USA) for PROX1. Tissues were incubated with beta-Catenin Antibody (Invitrogen, Thermo Fisher Scientific, Inc., Waltham, MA, USA; diluted to 1:500 = 5 μg/ml) for one hour at room temperature, and with Anti-human Prox1 Antibody (R&D Systems, Inc., Minneapolis, MN, USA; diluted to 1:1500 = 15 μg/ml) for overnight at room temperature. Samples of colon tissue and normal lymph node served as positive controls in each staining series (see Additional files 1 and 2). We also chose 13 whole tumor tissue blocks and corresponding lymph node metastases from the patient cohort to compare PROX1 expression in the tumor and its lymph node metastases.

### Evaluation of stainings

Cytoplasmic stainings of PROX1 and β-catenin were scored as negative (0), weakly positive (1), moderately positive (2), or strongly positive (3) according to staining intensity. Also, β-catenin membranous staining was evaluated. In the samples, where no membranous staining was seen, there was no cytoplasmic staining either. The highest score of each sample was considered representative for analysis. Scoring was performed by two independent investigators (K.S. and J.H.) without knowledge of clinical data and outcome. In case of differing scores, consensus score was discussed and determined.

### Statistical analyses

Categories of β-catenin and PROX1 were dichotomized for statistical purposes into low (scores 0–1) and high (scores 2–3). A three-class categorization was created to study these two tumor markers together: low (PROX1, and β-catenin low), moderate (either PROX1, or β-catenin high), and high (PROX1, and β-catenin high).

Associations between tumor marker expression and clinicopathological parameters were assessed by the Fischer’s exact-test or the linear-by-linear association test. The Kaplan-Meier method and log-rank test were used for survival analysis. The Bonferroni correction was used for multiple comparisons by dividing the probability level by the number of comparisons. The Spearman correlation coefficient with bootstrapped (1000 resamples, bias corrected) confidence intervals was calculated to find out correlations between PROX1 and β-catenin expression. Uni- and multivariate survival analyses were carried out by the Cox regression proportional hazard model adjusted for age, gender, stage, metastasized lymph node ratio (LNR) ≥/<20 % (cut-off ≥/<20 %), perivascular invasion, and postoperative adjuvant therapy. Since stage and LNR are internally correlated to each other, a combination variable was formed for multivariate analyses (see Table 6). Interaction terms were considered. The Cox model assumption of constant hazard ratios over time was tested. For each testable variable at a time, a time-dependent covariate was included separately. All variables fulfilled the assumption. Stage and lymph node ratio were combined into a single variable to simplify the model. A p-value <0.05 was considered significant and all tests were two-sided. Statistical analyses were computed with SPSS version 22.0 (IBM SPSS Statistics, version 22.0 for Windows/MAC; SPSS, Inc., Chicago, IL, USA, an IBM Company).

## Results

### Immunohistochemical staining

**Fig. 1:**
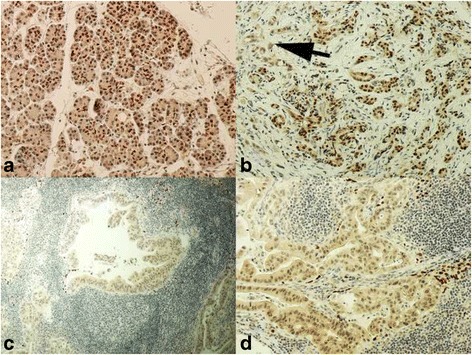
Immunohistochemical staining pattern of PROX1 in normal pancreas, in pancreatic ductal adenocarcinoma (PDAC), and in metastasized lymph node. **a** PROX1 staining pattern in normal pancreatic tissue. Distinct nuclear positivity to be seen. **b** PROX1 staining pattern in transitional zone of normal pancreas and cancerous tissue *(arrowhead)*. **c** and **d** PROX1 staining pattern in metastasized lymph node. No nuclear expression in cancer cells. Original magnification × 200 in (**a**, **b**, and **d**). Original magnification × 100 in C

PROX1 expression was cytoplasmic and evenly distributed with no distinctive membranous staining. Cytoplasmic staining was scored as described above. In normal pancreatic tissue apparent nuclear staining is present although all the nuclei are not stained. In two cancer tissue samples we saw staining of the nuclei, and the cytoplasmic staining scores in these samples were 1, and 3. In all the other cancer specimens nuclei were negative. In the whole tumor specimens, there was no nuclear staining in the metastases**;** only negative or weak cytoplasmic staining was present (Fig. 1).

**Fig. 2:**
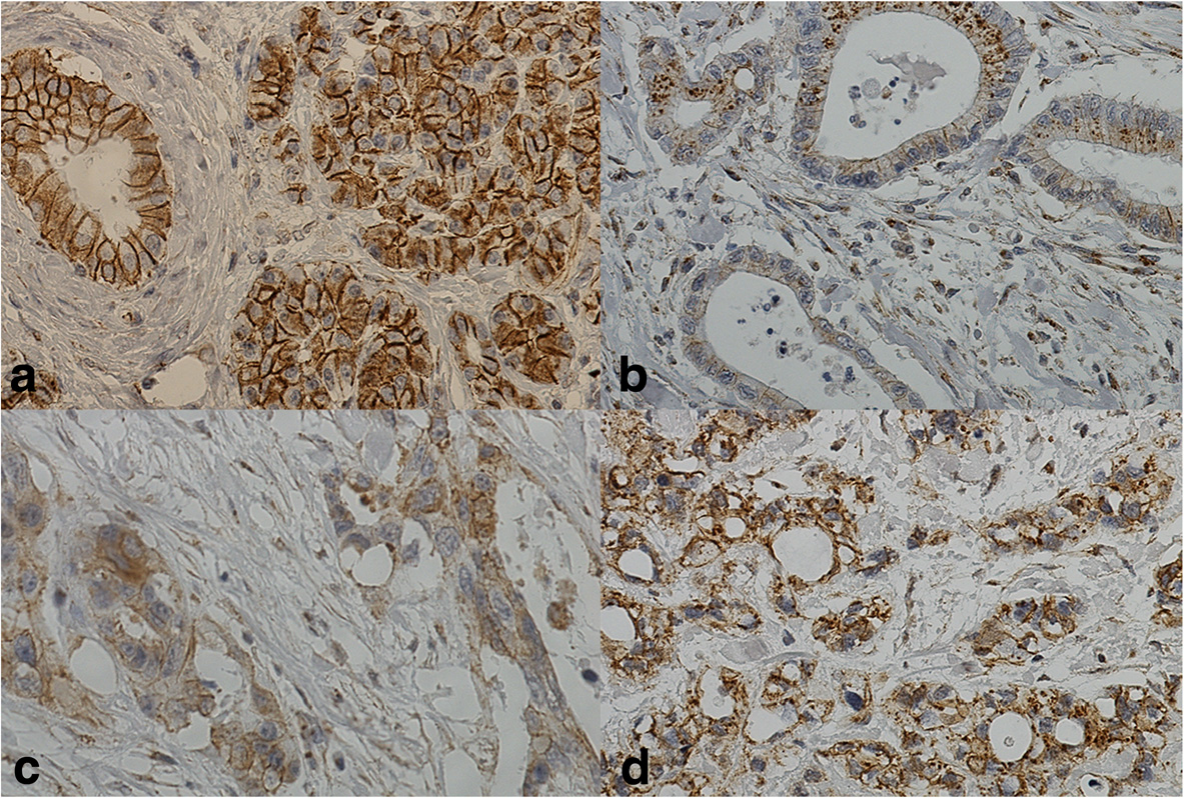
Immunohistochemical staining pattern of β-catenin in normal pancreas and in pancreatic ductal adenocarcinoma (PDAC). **a** β-catenin expression pattern in normal pancreatic tissue. Distinct membranous staining to be seen. **b** Weak cytoplasmic β-catenin expression positivity in PDAC with no membranous positivity. **c** Weak cytoplasmic β-catenin expression positivity in PDAC with some membranous positivity. **d** Moderate cytoplasmic β-catenin expression positivity in PDAC with distinct granular appearance. Original magnification × 400

β-catenin expression was distributed in the cell membrane and within the cytoplasm. Only in a few exceptions the staining was not uniform throughout the cell. With more intense membranous staining, also cytoplasmic staining was stronger. The cytoplasmic expression pattern showed two different types of staining; homogenous and granular. There was no distinct nuclear staining. Only three samples lacked membranous staining (Fig. 2). The membranous and cytoplasmic staining were very difficult to score separately. Because of this, cytoplasmic expression was used in statistical analyses.

**Fig. 3:**
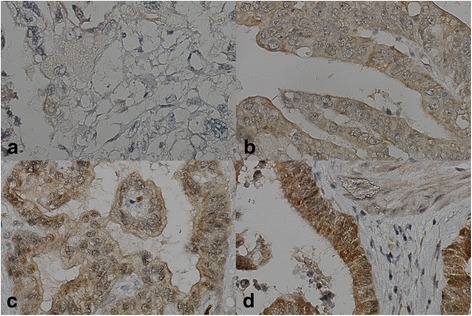
Immunohistochemical staining pattern of PROX1 in pancreatic ductal adenocarcinoma (PDAC). Representative images of PROX1 expression in PDAC. **a** negative, **b** weak, **c** moderate, and **d** strong cytoplasmic positivity without staining of the nuclei. Original magnification × 400

**Fig. 4:**
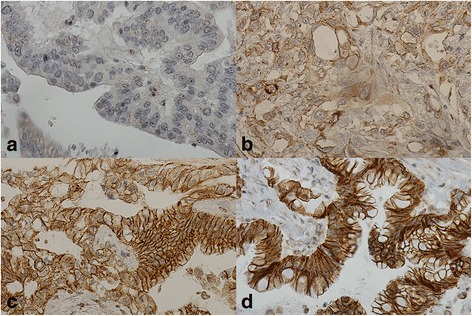
Immunohistochemical staining pattern of β-catenin in pancreatic ductal adenocarcinoma (PDAC). Representative images of β-catenin expression in PDAC. **a** negative cytoplasmic, **b** weak cytoplasmic and membranous positivity, **c** moderate cytoplasmic, and **d** strong cytoplasmic and positive membranous positivity. Original magnification × 400

PROX1 staining could be evaluated in 154 (99 %) specimens: 20 (13 %) showing negative, 60 (39 %) weak, 66 moderate, (43 %) and 8 (5 %) strong staining (Fig. 3). β-catenin cytoplasmic staining could be evaluated in 153 (98 %) specimens: 1 (1 %) showing negative, 52 (34 %) weak, 63 (41 %) moderate, and 37 (24 %) strong staining (Fig. 4). Combined PROX1 and β-catenin expression was evaluated in 152 (97 %) tumors: 38 (25 %) low, 56 (37 %) moderate, and 58 (38 %) high expression pattern.

### Association between PROX1 and β-catenin expression and clinicopathological variables

There was a statistically significant association between PROX1 expression and age; patients in the low PROX1 expression group were younger than in the high PROX1 expression group (*p* = 0.038). PROX1 expression did not correlate with gender, stage, LNR, histological grade, perineural, or perivascular invasion (Table 1).

**Table 1:** Association of clinicopathological parameters and PROX1 expression

PROX1 expression			
	Low (0–1)	High (2–3)	
n(%)	80 (51.9)	74 (48.1)	*p*-value
Age, years			
< 65	46 (57.5)	30 (40.5)	0.038
≥ 65	34 (42.5)	44 (59.5)	
Gender			
Male	45 (56.3)	40 (54.1)	0.871
Female	35 (43.7)	34 (45.9)	
T			
1	5 (6.3)	7 (9.5)	0.274
2	18 (22.5)	22 (29.7)	
3	56 (70.0)	43 (58.1)	
4	1 (1.3)	2 (2.7)	
N			
0	23 (28.8)	25 (33.8)	0.602
1	57 (71.2)	49 (66.2)	
Stage (WHO)			
IA	4 (5.0)	5 (6.8)	0.550
IB	8 (10.0)	10 (13.5)	
IIA	11 (13.8)	9 (12.2)	
IIB	56 (70.0)	48 (64.9)	
III	1 (1.3)	2 (2.7)	
Lymph node ratio			
< 20 %	57 (71.3)	60 (83.3)	0.086
≥ 20 %	23 (28.7)	12 (16.7)	
Missing		2	
Grade			
1	10 (14.7)	12 (19.0)	0.543
2	47 (69.1)	42 (66.7)	
3	11 (16.2)	9 (14.3)	
Missing	12	11	
Perineural invasion			
Yes	49 (73.1)	51 (82.3)	0.291
No	18 (26.9)	11 (17.7)	
Missing	13	12	
Perivascular invasion			
Yes	24 (36.9)	19 (32.2)	0.706
No	41 (63.1)	40 (67.8)	
Missing	15	15	

Fischer exact-test was used for 2×2 tables and linear-by-linear association test for tables with more than two rows. Missing data excluded from the analyses

Patients with low β-catenin expression showed a significant association with higher tumor histological grade compared to patients with high expression (*p* = 0.025). No significant association was found between β-catenin and age, gender, stage, LNR, perineural, or perivascular invasion (Table 2).

**Table 2:** Association of clinicopathological parameters and β-catenin expression

β-catenin expression			
	Low (0–1)	High (2–3)	
n(%)	53 (34.6)	100 (65.4)	*p*-value
Age, years			
< 65	28 (52.8)	48 (48.0)	0.613
≥ 65	25 (47.2)	52 (52.0)	
Gender			
Male	32 (60.4)	52 (52.0)	0.394
Female	21 (39.6)	48 (48.0)	
T			
1	3 (5.7)	8 (8.0)	0.602
2	14 (26.4)	26 (26.0)	
3	34 (64.2)	65 (65.0)	
4	2 (3.8)	1 (1.0)	
N			
0	15 (28.3)	32 (32.0)	0.714
1	38 (71.7)	68 (68.0)	
Stage (WHO)			
IA	3 (5.7)	6 (6.0)	0.590
IB	6 (11.3)	12 (12.0)	
IIA	5 (9.4)	14 (14.0)	
IIB	37 (69.8)	67 (67.0)	
III	2 (3.8)	1 (1.0)	
Lymph node ratio			
< 20 %	40 (75.5)	77 (78.6)	0.687
≥ 20 %	13 (24.5)	21 (21.4)	
Missing		2	
Grade			
1	5 (10.9)	17 (19.8)	0.025
2	29 (63.0)	60 (69.8)	
3	12 (26.1)	9 (10.5)	
Missing	7	14	
Perineural invasion			
Yes	34 (77.3)	66 (77.6)	1.000
No	10 (22.7)	19 (22.4)	
Missing	9	15	
Perivascular invasion			
Yes	19 (43.2)	24 (30.0)	0.169
No	25 (56.8)	56 (70.0)	
Missing	9	20	

Fischer exact-test was used for 2×2 tables and linear-by-linear association test for tables with more than two rows. Missing data excluded from the analyses

There was no correlation between combined PROX1 and β-catenin expression and age, gender, stage, histological grade, LNR, perineural, or perivascular invasion (Table 3). PROX1 and β-catenin expression correlated with each other (Spearman correlation coefficient = 0,371; 95 % CI 0.24–0.50; *p* < 0.001).

**Table 3:** Association of clinicopathological parameters and β-catenin and PROX1 expression combined

	Low	Moderate	High	
n (%)	38 (25.0)	56 (36.8)	58 (38.2)	*p*-value
Age, years				
< 65	21 (55.3)	32 (57.1)	23 (39.7)	0.121
≥ 65	17 (44.7)	24 (42.9)	35 (60.3)	
Gender				
Male	23 (60.5)	31 (55.4)	30 (51.7)	0.409
Female	15 (39.5)	25 (44.6)	28 (48.3)	
T				
1	2 (5.3)	4 (7.1)	5 (8.6)	0.343
2	10 (26.3)	12 (21.4)	18 (31.0)	
3	25 (65.8)	39 (69.6)	34 (58.6)	
4	1 (2.6)	1 (1.8)	1 (1.7)	
N				
0	10 (26.3)	17 (30.4)	20 (34.5)	0.436
1	28 (73.7)	39 (69.6)	38 (65.5)	
Stage (WHO)				
IA	2 (5.3)	3 (5.4)	4 (6.9)	0.412
IB	4 (10.5)	6 (10.7)	8 (13.8)	
IIA	4 (10.5)	7 (12.5)	8 (13.8)	
IIB	27 (71.1)	39 (69.6)	37 (63.8)	
III	1 (2.6)	1 (1.8)	1 (1.7)	
Lymph node ratio				
< 20 %	29 (76.3)	38 (67.9)	49 (87.5)	0.138
≥ 20 %	9 (23.7)	18 (32.1)	7 (12.5)	
Missing			2	
Grade				
1	4 (12.5)	7 (14.0)	11 (22.4)	0.059
2	20 (62.5)	36 (72.0)	33 (67.3)	
3	8 (25.0)	7 (14.0)	5 (10.2)	
Missing	6	6	9	
Perineural invasion				
Yes	25 (73.5)	32 (76.2)	42 (80.8)	0.438
No	9 (26.5)	10 (23.8)	10 (19.2)	
Missing	4	14	6	
Perivascular invasion				
Yes	13 (38.2)	17 (42.5)	13 (26.5)	0.247
No	21 (61.8)	23 (57.5)	36 (73.5)	
Missing	4	16	9	

Linear-by-linear association test was used here

### Survival analysis

**Fig. 5:**
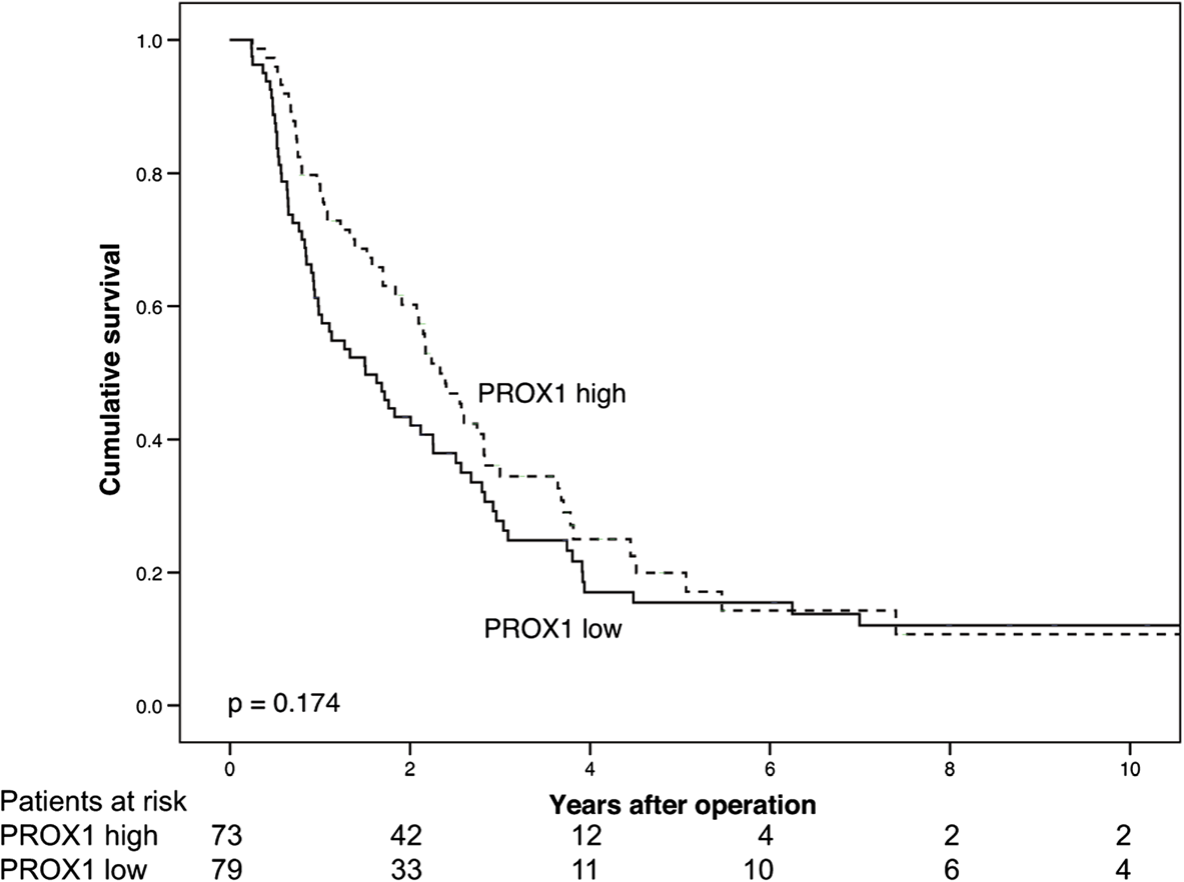
Low PROX1 expression suggests a poor prognosis in pancreatic ductal adenocarcinoma. Cancer-specific survival analysis according to the Kaplan-Meier method for PROX1 expression in pancreatic ductal adenocarcinoma

**Fig. 6:**
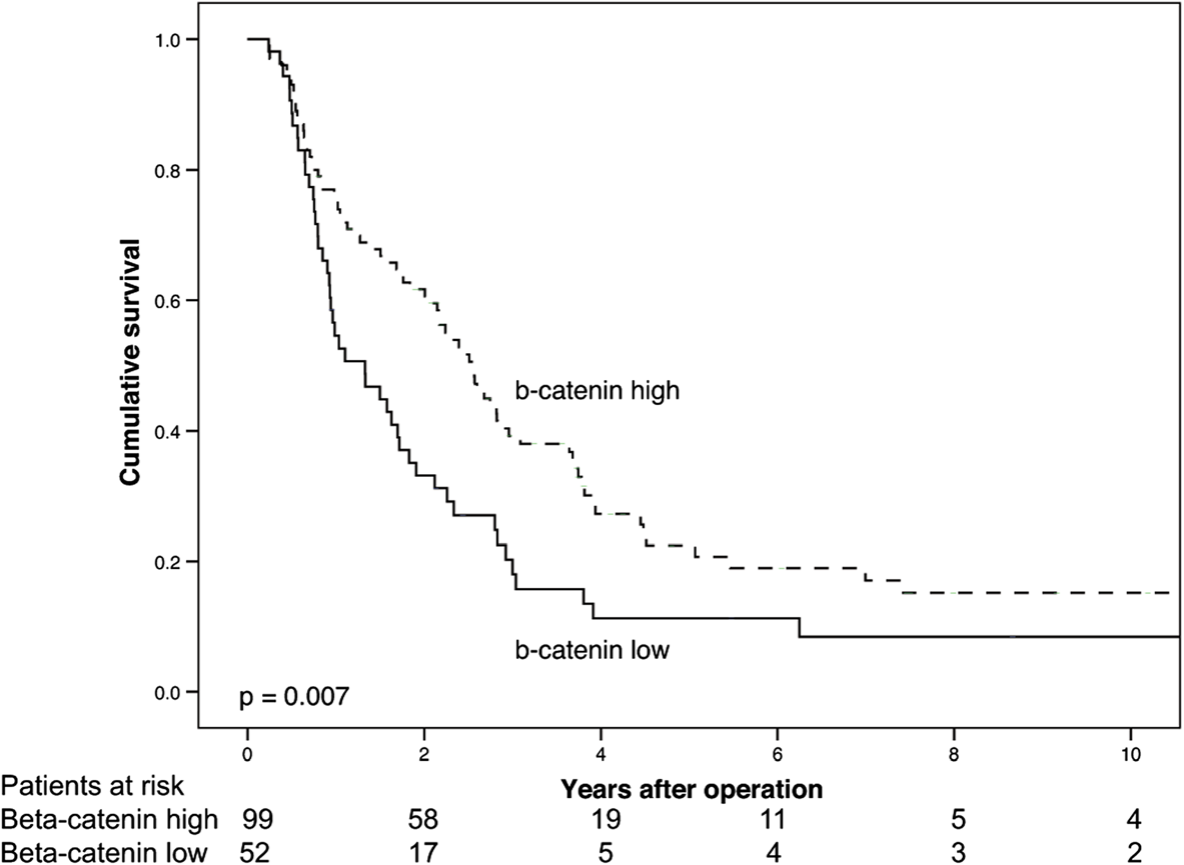
Low β-catenin expression is a marker of poor prognosis in pancreatic ductal adenocarcinoma. Cancer-specific survival analysis according to the Kaplan-Meier method for β-catenin expression in pancreatic ductal adenocarcinoma

Five-year cancer-specific survival (CSS) was not significantly different for PDAC patients with low PROX1 expression compared to those with high expression (log-rank, *p* = 0.174, Fig. 5). Five-year CSS was 15.5 % (95 % CI 6.7–24.3 %) for patients with low PROX1 expression, and 20.0 % (95 % CI 9.2–30.8 %) when PROX1 expression was high (Table 4). PDAC patients with low β-catenin expression showed significantly poorer CSS than those patients with high expression (log-rank, *p* = 0.007, Fig. 6). Five-year CSS for PDAC patients with low β-catenin expression was 11.3 % (95 % CI 2.1–20.5 %), and 22.4 % (95 % CI 13.0*–*31.8 %) for those with high expression (Table 4).

**Table 4:** Cancer-specific survival (CSS) for pancreatic ductal adenocarcinoma patients by PROX1 and β-catenin expression

	Five-year CSS		Two-year CSS	
	CSS (%)	95 % CI	CSS (%)	95 % CI
PROX1 expression				
Low	15.5	6.7–24.3	43.4	32.2–54.6
High	20.0	9.2–30.8	60.2	49.8–71.6
β-catenin expression				
Low	11.3	2.1–20.5	33.1	20.1–46.1
High	22.4	13.0–31.8	61.6	51.8–71.4
Combined expression				
Low	10.3	−0.7–21.3	33.2	17.6–48.8
Moderate	18.7	9.9–29.5	48.2	34.8–61.6
High	21.3	8.1–34.5	66.7	54.3–79.1

Abbrevations: *CSS* cancer-specific survival, *CI* confidence interval. Combined expression refers to combined expression of PROX1 and β-catenin

**Fig. 7:**
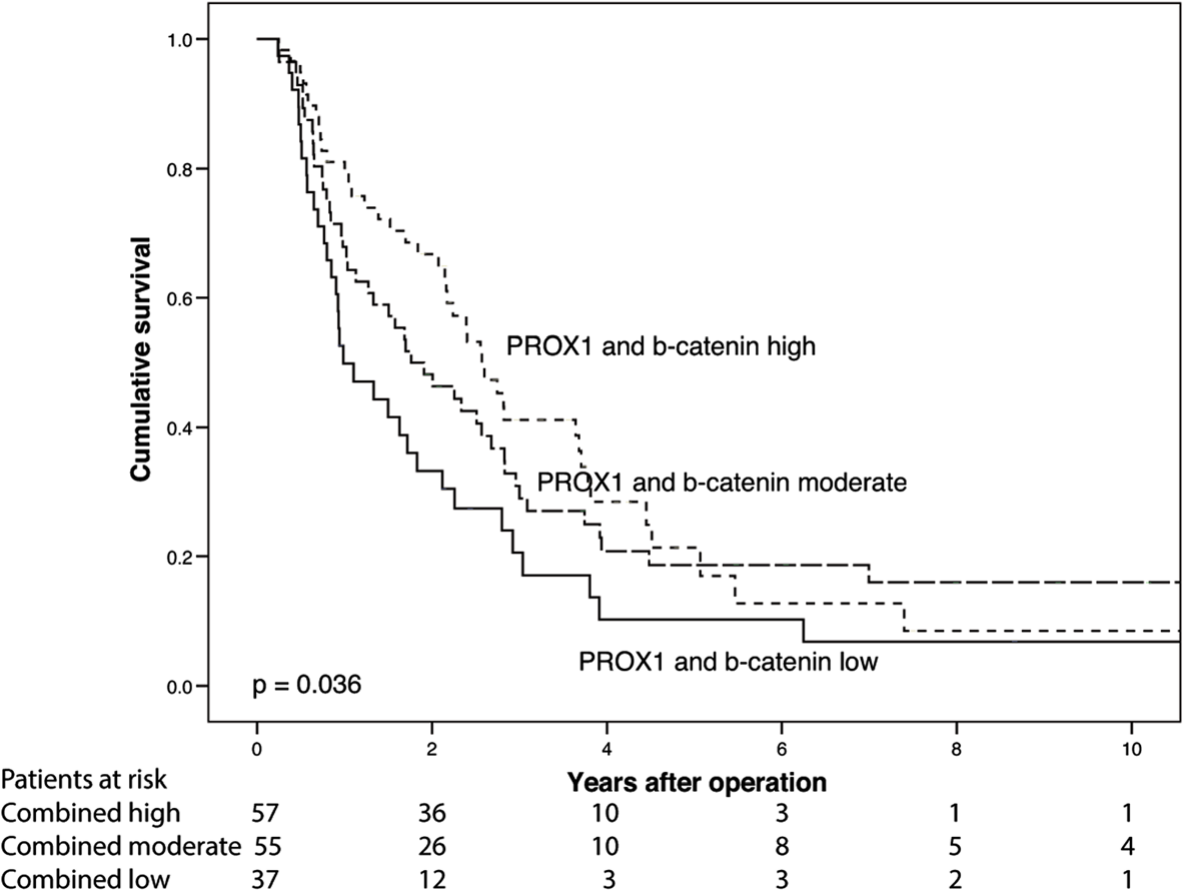
Concomitant positivity by β-catenin and PROX1. Cancer-specific survival analysis according to the Kaplan-Meier method for combined expression of β-catenin and PROX1. A categorization of three classes was created to study the two markers together: low (β-catenin, and PROX1, low), moderate (either β-catenin, or PROX1 high), and high (β-catenin, and PROX1 high)

Combined expression of PROX1 and β-catenin showed significantly poorer CSS for PDAC patients with low compared to high expression (*p* = 0.013). Between patients with moderate and low expression (*p* = 0.092), or with moderate and high expression (*p* = 0.435) no significant difference in CSS was seen (Fig. 7). Five-year CSS for patients with low combined expression was 10.3 % (95 % CI −0.7–21.3 %), with moderate combined expression 18.7 % (95 % CI 9.9–29.5 %), and with high combined expression 21.3 % (95 % CI 8.1–34.5 %) (Table 4).

In univariate analyses high β-catenin expression associated significantly with lower risk of death from PDAC (HR = 0.61, 95 % CI 0.42–0.88; *p* = 0.008). High PROX1 expression seemed to reduce the risk of death from PDAC, but this result just failed to be statistically significant in univariate analysis (HR = 0.71, 95 % CI 0.49–1.01; *p* = 0.053). With PROX1 and β-catenin, combined high expression showed lower risk of death from PDAC (HR = 0.52, 95 % CI 0.33–0.83; *p* = 0.006). With moderate combined expression the risk of death from PDAC was not statistically significant (HR = 0.69, 95 % CI 0.44–1.08; *p* = 0.103). Other prognostic variables in univariate analyses were stage, lymph node positivity, perivascular invasion, and postoperative adjuvant therapy (Table 5).

**Table 5:** Cox univariate analysis of relative risk of death from pancreatic ductal adenocarcinoma by β-catenin and PROX1 expression (*n* = 156)

Covariate	*n*	HR	95 % CI	*p*-value
PROX1 expression				
Low	80	1.00		1.000
High	74	0.70	0.49–1.00	0.053
Missing	2			
β-catenin expression				
Low	53	1.00		1.000
High	100	0.61	0.42–0.88	0.008
Missing	3			
Combined PROX1 and β-catenin expression				
Low	38	1.00		1.000
Moderate	56	0.69	0.22–1.01	0.103
High	58	0.52	0.33–0.83	0.006
Missing	4			
Age at operation				
< 65	77	1.00		1.000
≥ 65	79	1.06	0.74–1.50	0.752
Gender				
Male	86	1.00		1.000
Female	70	0.93	0.65–1.32	0.668
T				
1	12	1.00		1.000
2	40	1.02	0.47–2.24	0.957
3	101	1.65	0.80–3.41	0.178
4	3	4.09	1.07–15.66	0.040
N				
0	48	1.00		1.000
1	108	1.80	1.20–2.70	0.004
Grade				
1	22	1.00		1.000
2	90	1.14	0.66–1.96	0.644
3	21	2.05	1.04–4.02	0.038
Missing	23			
Stage				
IA, and IB	27	1.00		1.000
IIA, and IIB	126	2.01	1.19–3.41	0.009
III	3	5.23	1.49–18.34	0.010
Stage and LNR				
IA, IB, and IIA	47	1.00		1.000
IIB, III and LNR <20 %	73	1.50	0.97–2.31	0.071
IIB, III and LNR >20 %	36	3.12	1.91–5.11	<0.001
Perivascular invasion				
No	43	1.00		1.000
Yes	83	1.89	1.27–2.83	0.002
Missing	30			
Perineural invasion				
No	30	1.00		1.000
Yes	101	1.44	0.90–2.31	0.126
Missing	25			
Postoperative adjuvant therapy				
No	75	1.00		1.000
Yes	79	0.65	0.45–0.92	0.016
Missing	2			

*Abbreviations*: *HR* hazard ratio, *CI* confidence interval, *LNR* metastasized lymph node ratio. Stage and LNR covariate was formed to cover both in multivariate analysis

In multivariate analyses adjusted for age, gender, stage, LNR, perivascular invasion, and adjuvant therapy high β-catenin expression remained statistically significant for better prognosis (HR = 0.54, 95 % CI 0.35–0.82; *p* = 0.004), and high PROX1 expression was also statistically significant (HR 0.63, 95 % CI 0.42–0.95; *p* = 0.026). The combined high expression of β-catenin and PROX1 remained statistically significant (HR 0.46, 95 % CI 0.28–0.76; *p* = 0.002) (Table 6).

**Table 6:** Cox multivariate analysis of relative risk of death from pancreatic ductal adenocarcinoma by β-catenin and PROX1 expression

β-catenin			PROX1			Combined		
β-catenin expression	HR (95 % CI)	*P*-value	PROX1 expression	HR (95 % CI)	*P*-value	β-catenin and PROX expression	(HR 95 % CI)	*P*-value
Low	1.00		Low	1.00		Low	1.00	
High	0.54 (0.35–0.82)	0.004	High	0.63 (0.42–0.95)	0.026	Moderate	0.61 (0.36–1.03)	0.063
						High	0.46 (0.28–0.76)	0.002

Abbreviations *CI* Confidence interval, *HR* Hazard ratio. Multivariate analysis included adjustment for age, gender, stage (IA-IIA, IIB and III) and lymph node ratio (≥/< 20 %), postoperative adjuvant therapy, and perivascular invasion

## Discussion

We here show that high tissue expression of PROX1 and β-catenin independently predict better prognosis in PDAC.

PROX1 expression is vital for pancreatic development. Loss of PROX1 in the pancreas leads to remarkable size reduction [31], and premature acinar cell differentiation and increased ductal cell proliferation [32]. Schneider et al. reported in 2006 that pancreatic cancer cells express less PROX1 mRNA than normal exocrine pancreatic cells [29]. They noticed that PROX1 gene expression levels were lower in patients with survival less than 6 months. Our study shows a similar tendency by immunohistochemistry although the difference in survival was not significant. To our knowledge, no prognostic studies on PROX1 protein expression in PDAC have been reported so far.

Increased PROX1 expression has been discovered to be associated with poor prognosis in CRC although it was not an independent prognostic factor in multivariate analysis [33]. These results are opposite to our results in PDAC. In CRC, high PROX1 expression was associated with high tumor grade. This finding was not confirmed in our study. PROX1 is required for the formation of lymphatic vasculature [20], and overexpression of PROX1 in blood endothelial cells induces lymphatic endothelial cell gene expression [34]. However, Schneider et al. suggested that active lymphangiogenesis is not needed for lymphovascular spread in pancreatic cancer [30]. Recent data shows that positive PROX1 expression correlates with positive lymph node metastases in CRC and gastric cancer [35, 36]. It remains unclear whether the downregulation of PROX1 expression enhances the lymphatic metastatic spread of pancreatic ductal adenocarcinoma.

We evaluated the staining of PROX1 in the cytoplasm, whereas in the previous studies of CRC, hepatocellular carcinoma (HCC), and gliomas, only the staining in tumor cell nuclei were evaluated [24, 28, 33]. In a recent study of gastric cancer, also cytoplasmic PROX1 expression by IHC was evaluated and it correlated with mRNA amplification [37]. We noted nuclear staining only in two tumor specimens. However, nuclear staining is present in the normal pancreas. At some point, the nuclear expression decreases, and in cancerous tissue, only cytoplasmic expression is left. These findings suggest that PROX1 may not function as an active transcription factor in PDAC. The role of cytoplasmic PROX1 expression has been studied in papillary thyroid cancer (PTC) by Choi et al. [38]. They discovered that PROX1 becomes inactivated through mRNA downregulation by aberrant NOTCH signaling, and cytoplasmic mislocalization of PROX1 increases protein stability in PTC cells. In addition, restoration of PROX1 impaired tumor formation and diminished invasiveness of PTC cells.

Whether the downexpression in the nuclei results from the evolved pancreatic cancer, or results in pancreatic cancer formation, remains unknown. Because of the limitations of IHC, we can only speculate, whether cytoplasmic PROX1 in pancreatic tumor tissue is in active or inactive form. The main remaining question is what the role of cytoplasmic PROX1 expression is and what the signal is that leads to relocation to the cytoplasm [38]. This question needs further studies to clarify the role of cytoplasmic PROX1 expression in PDAC. Our results are in line with the findings of PROX1 expression earlier discovered in PDAC, but also show the different nature of CRC and PDAC.

In the normal pancreas β-catenin expression is predominantly localized in the membrane of ductal cells. In pancreatic cancer, down-regulation of membrane expression and increased cytoplasmic expression are seen [11–14]. In our series, mainly uniform, granular cytoplasmic and membranous staining were seen, but there were only three specimens lacking membranous staining.

A few studies have reported that reduced or abnormal membranous β-catenin expression predicts poor prognosis of PDAC patients [11, 13]. Wang et al. found no prognostic impact of β-catenin cytoplasmic expression in PDAC [14]. Qiao et al. showed that reduced membranous and positive cytoplasmic expression of β-catenin associated with poorer survival in PDAC during one-year follow-up [12]. These results differ from ours but the follow-up times in previous studies are only one or two years, and the patient cohorts have been small (*n* = 43–48). In none of those studies nuclear positivity was reported. In a few studies reduced β-catenin expression correlated with tumor dedifferentiation, but the prognostic significance was not investigated [9, 39]. However, the controversy remains as in gene array analysis it has been demonstrated, that inhibition of Wnt/β-catenin signaling pathway blocks proliferation and induces apoptosis of cultured PDAC cells [10]. Also, increased β-catenin expression and protein levels have been reported in pancreatic tumors [7]. There is a need for further studies to validate the role of β-catenin expression in PDAC as reviewed by Morris et al. [40]. Our study shows by IHC that β-catenin expression in PDAC is both membranous and cytoplasmic with no distinct nuclear staining, and high β-catenin expression predicts better prognosis.

The combination of PROX1 and β-catenin expression was created, because they have been linked to the same signaling pathway and their activation/expression is increased in CRC [15, 23, 33]. Furthermore, Yu et al. showed recently in CRC that β-catenin-PROX1 signaling axis is regulated by a transcriptional coactivator deleted in breast cancer (DBC1) [41]. They concluded that DBC1 acts as a positive regulator and as a key factor of β-catenin-PROX1 signaling axis in CRC progression. We demonstrate by IHC that both PROX1 and β-catenin expression are decreased in PDAC patients, and their expression are correlated significantly. These results were opposite from those in CRC. Whether β-catenin and PROX1 function in the same, yet opposite, way, remains unclear. However, we did not find any significant prognostic effect with combined PROX1 and β-catenin expression compared to β-catenin expression alone. Further analyses are required to examine the activity of Wnt/β-catenin/PROX1 signaling pathway in PDAC. In order to thoroughly analyze the effect of PROX1 and β-catenin IHC expression on prognosis, a multi-center study with larger patient cohorts would be needed.

The TMA technique allows analysis of large patient cohorts. On the other hand, smaller areas of the tumors are evaluated compared to whole tissue sections. By taking cores from different parts of the tumor, possible sampling error can be diminished. Only less than 2 % of the specimens were lost in this patient cohort because of technical reasons. The strength of this study is a quite large patient cohort with long follow-up time. Unfortunately, due to the long period of data collecting, some of the crucial clinicopathological parameters were not available. Also, one of the weaknesses of the study is the lack of knowledge of the reliable resection margin status (R0/R1), which is known to be an important prognostic factor [42]. This results from the fact that our study is retrospective, and only in the last few years clinicians and pathologists have drawn enough attention to this important matter. All histological specimens were re-evaluated and only ductal adenocarcinomas were included in the study.

## Conclusion

We show that high tissue expression of PROX1 and β-catenin, both independently, predict better prognosis in PDAC. PROX1 expression is not seen in the nuclei of PDAC cells, but in the cytoplasm. β-catenin expression localizes both to the cytoplasm and to the cell membrane. To our knowledge, this is the first report on the prognostic value of PROX1 protein expression in PDAC.

## Abbreviations

APC, adenomatous polyposis coli; CI, confidence interval; CRC, colorectal cancer; CSS, cancer-specific survival; CTNNB1, protein β-catenin encoding gene; DBC1, deleted in breast cancer; HCC, hepatocellular carcinoma; HR, hazard ratio; IFN-γ, interferone γ; IHC, immunohistochemistry; LNR, lymph node ratio; PDAC, pancreatic ductal adenocarcinoma; PROX1, prospero homeobox protein 1; PTC, papillary thyroid cancer; TCF/LEF, T-cell factor/lymphoid enhancer factor; TMA, tissue microarray

## Additional files

Additional file 1: Positive control of immunohistochemical expression of PROX1 in colon tissue. (TIF 3631 kb) Additional file 2: Positive control of immunohistochemical expression of β-catenin in colon tissue. (TIF 3631 kb)
