# Supplementary figures and images for: PROX1 and β-catenin are prognostic markers in pancreatic ductal adenocarcinoma
Source: BMC Cancer. 2016 Jul 13;16:472. doi: 10.1186/s12885-016-2497-5 (PMC4944261; doi:10.1186/s12885-016-2497-5)

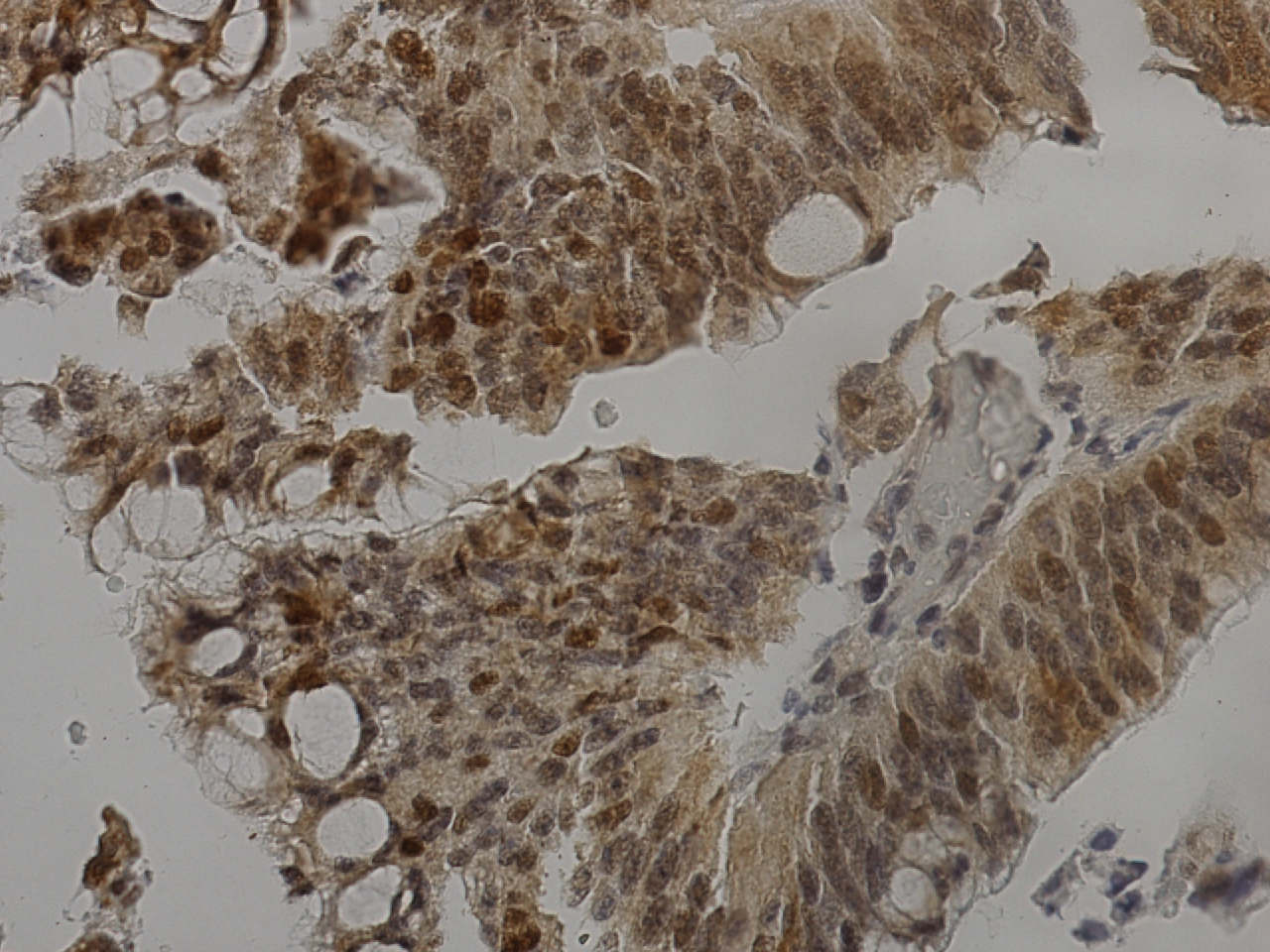

Supplement: Additional file 1: — Positive control of immunohistochemical expression of PROX1 in colon tissue. (TIF 3631 kb) [file 12885_2016_2497_MOESM1_ESM.tif]

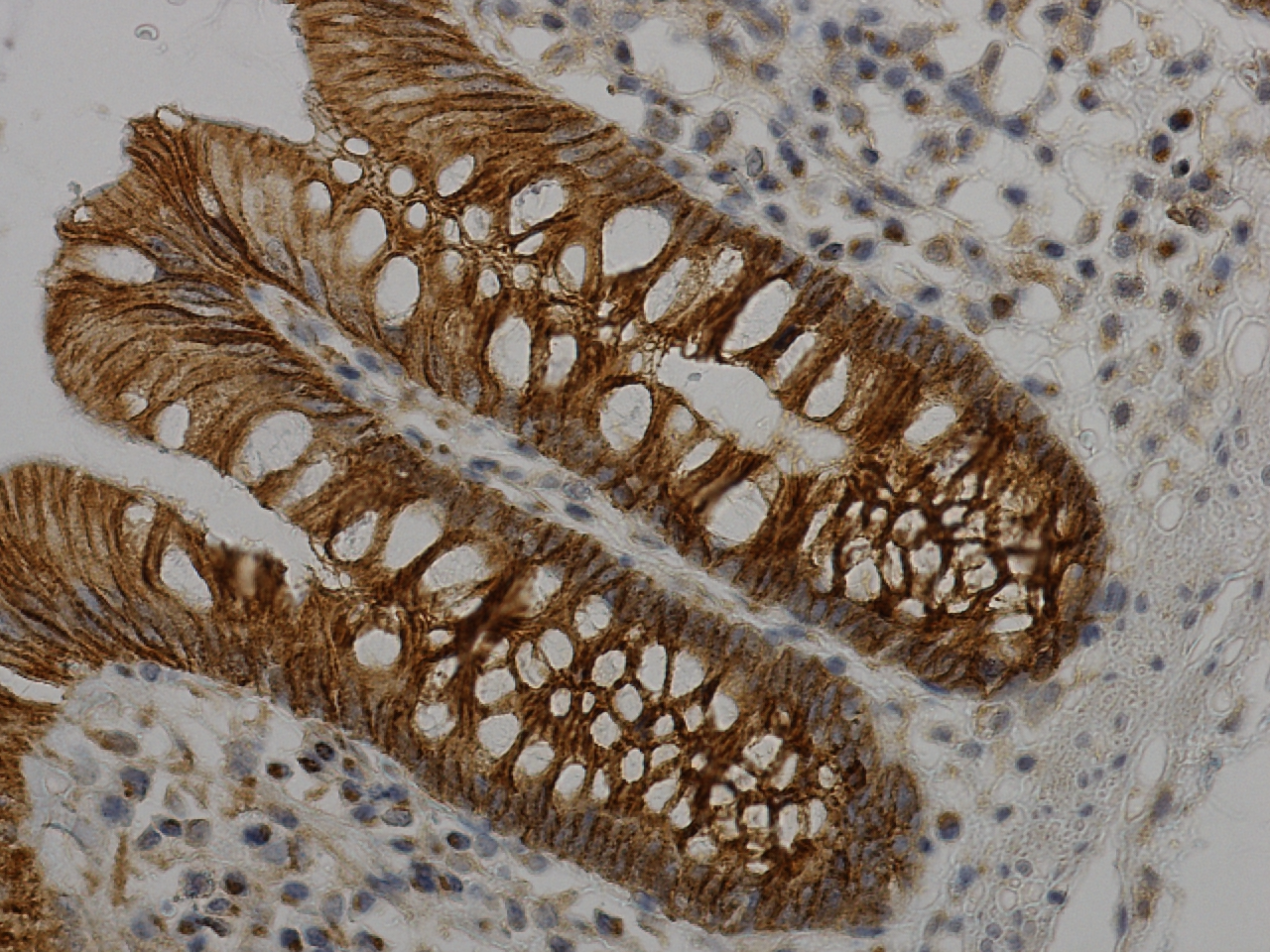

Supplement: Additional file 2: — Positive control of immunohistochemical expression of β-catenin in colon tissue. (TIF 3631 kb) [file 12885_2016_2497_MOESM2_ESM.tif]
